# Management of Antiretroviral Therapy with Boosted Protease Inhibitors—Darunavir/Ritonavir or Darunavir/Cobicistat

**DOI:** 10.3390/biomedicines9030313

**Published:** 2021-03-18

**Authors:** Ruxandra-Cristina Marin, Tapan Behl, Nicoleta Negrut, Simona Bungau

**Affiliations:** 1 Department of Pharmacy, Faculty of Medicine and Pharmacy, University of Oradea, 410028 Oradea, Romania; rcfhm@yahoo.com; 2 Chitkara College of Pharmacy, Chitkara University, Punjab 140401, India; tapanbehl31@gmail.com; 3 Department of Psycho-Neuroscience and Recovery, Faculty of Medicine and Pharmacy, University of Oradea, 410028 Oradea, Romania; lnm_n10@yahoo.com

**Keywords:** antiretroviral therapy (ART), antiretrovirals (ARVs), boosted protease inhibitor (PI), darunavir (DRV), ritonavir (RTV), cobicistat (COBI), HIV/AIDS

## Abstract

A major challenge in the management of antiretroviral therapy (ART) is to improve the patient’s adherence, reducing the burden caused by the high number of drugs that compose the treatment regimens for human immunodeficiency virus positive (HIV+) patients. Selection of the most appropriate treatment regimen is responsible for therapeutic success and aims to reduce viremia, increase the immune system response capacity, and reduce the incidence rate and intensity of adverse reactions. In general, protease inhibitor (PI) is one of the pillars of regimens, and darunavir (DRV), in particular, is frequently recommended, along with low doses of enzyme inhibitors as cobicistat (COBI) or ritonavir (RTV), by the international guidelines. The potential of clinically significant drug interactions in patients taking COBI or RTV is high due to the potent inhibitory effect on cytochrome CYP 450, which attracts significant changes in the pharmacokinetics of PIs. Regardless of the patient or type of virus, the combined regimens of DRV/COBI or DRV/RTV are available to clinicians, proving their effectiveness, with a major impact on HIV mortality/morbidity. This study presents current information on the pharmacokinetics, pharmacology, drug interactions, and adverse reactions of DRV; it not only compares the bioavailability, pharmacokinetic parameters, immunological and virological responses, but also the efficacy, advantages, and therapeutic disadvantages of DRV/COBI or DRV/RTV combinations.

## 1. Introduction

The human immunodeficiency virus (HIV), responsible for the development of acquired human immunodeficiency syndrome (AIDS), is today one of the most serious public health challenges that humanity has to deal with [1]. Nearly 40 million people worldwide are infected with HIV, and global efforts are being made to reduce the number of new infections and to give all patients access to antiretroviral treatment/therapy (ART). Aware of their condition, people infected with HIV take ART daily, thus often managing to keep a low or even undetectable viral load. This way, their life expectancy has increased significantly, and they can live a normal life.

Another major benefit of ART is the possibility of preventing virus transmission, as this therapy manages to significantly reduce the number of maternal–fetal transmissions [2]. Highly active ART (HAART) has significantly reduced viremia, increased the body defense capacity, improved general condition, reduced the number of opportunistic infections, and prolonged survival, but with potentially significant side effects, reported since 1997, especially in the first generations of antiretrovirals (ARVs) [3,4].

Patients with HIV infection may face increased risk of cardiovascular disease, neurocognitive disorders, liver disease, cancer, and bone demineralization. These comorbidities are not only attributed to the disease per se, but are considered, in some cases, side effects of ART. Comorbidities can also be caused by continued viral replication in so-called “sanctuaries,” or they can be consequences of chronic HIV inflammation. Nowadays, only a third of deaths among people infected with HIV are caused by the natural course of the disease. The rest of the deaths occur due to comorbidities, complications induced by drug interactions and toxicity of ART [5].

ART can inhibit viral replication, controlling the infection and thus prolonging the patient’s life. Moreover, people infected with HIV can lead a normal life for decades. However, even patients who initially responded well to the treatment may experience moments or periods of therapeutic failure. This failure can be caused by many factors: low/lower patient adherence, too low potency of the drug, viral resistance, cellular characteristics, and pharmacokinetic considerations [6].

There is an apparently large number of medicinal substances (30 molecules) available to HIV infected patients. Treatment regimens always include combinations of ARV drugs [7]. The treatment history is different for each patient. In the majority of cases, it is proven that none of the patients will remain on the same regimen over time. The more often these regimens change, the older the patient becomes (“therapeutically” speaking), and the lower the virological response. Thus, it is imperative that those who set up the regimens know exactly the concentration of drug substance needed to suppress HIV replication without generating resistance or toxicity problems [7].

It is known that HIV has the great ability to make genetic mutations. There are significant zonal differences in the type of virus, which requires in-depth study related to its evolution in the body, depending on the geographical region and genetics of the infected population.

This study aims to provide the main information on the topic related to ART usage in HIV-infected patients, especially regarding those in whose regimen a protease inhibitor (PI)—namely darunavir (DRV)—is introduced in combination with an enzyme inhibitor (a booster), which increases the ARV effect of PI. The pharmacokinetic of the PIs is significantly influenced when combined with ritonavir (RTV) or cobicistat (COBI) (both are boosters); therefore, a detailed analysis of changes in pharmacokinetic parameters is required in patients with this regimen. Management of ART involves choosing the best treatment regimen for the therapeutic success (by lowering viremia, increasing CD4 lymphocyte levels, reducing the frequency and intensity of ARV medication, avoiding toxicity, etc.). The article reviews information from the literature relevant to the topic. At this time, there is not much comparative data between the advantages and disadvantages of using one inhibitor or another, or to strictly compare the values of boosted pharmacokinetic parameters.

## 2. Antiretroviral (ARV) Medication

The main classes of ARV drugs comprise 30 molecules active against HIV-1 as follows: nucleoside or nucleotide reverse transcriptase inhibitors (NRTIs), non-nucleoside reverse transcriptase inhibitors (NNRTIs), integrase inhibitors, PIs, and pharmacokinetic enhancers (boosters). In addition, there are new fixed dose combinations between various molecules, designed to improve administration and thus achieve adherence:

- fixed dual combinations—emtricitabine/tenofovir, COBI/DRV, lamivudine/zidovudine, lopinavir/RTV, and abacavir/lamivudine;
- fixed triple combinations—abacavir/lamivudine/zidovudine and abacavir/lamivudine/dolutegravir;
- fixed quadruple combinations—elvitegravir/COBI/emtricitabine/tenofovir.

### 2.1. Human Immunodeficiency Virus (HIV) Protease

In the life cycle of HIV, the protease is an essential element for viral maturation. HIV protease is a homo dimeric aspartyl protease, each monomer being composed of 99 amino acid residues with a catalytic aspartic acid (Asp) at position 25. HIV-1 protease cleaves the precursor of Gag (Gag) and Gag-Pol polyproteins encoded by the HIV genome-1 at nine processing sites to produce mature active proteins. Pol-polyproteins are first cleaved from Gag-Pol polyproteins and then further digested into protease, reverse transcriptase (p51), H ribonuclease (RNase H) (p15), and integrase [8].

The active site is not fully exposed, being covered by two flaps, which must be opened to allow the substrates to access it. HIV-1 protease activity can be inhibited by blocking its active site. The indispensable role of HIV protease in viral maturation makes it a major target of ARV drugs. A large number of HIV protease protein structures have facilitated the formulation of new and improved inhibitors [9].

### 2.2. Protease Inhibitors (PIs)

The first PI approved by the United States was RTV (1996), shortly followed by indinavir (1996), nelfinavir (1997), saquinavir (1997), amprenavir (1999), lopinavir/RTV (2000), atazanavir (2003), fosamprenavir (2003), tipranavir (2005), and DRV (2006). The potency of these agents is similar, the choice of one of them in therapy is made taking into account the pharmacokinetics (regardless of how many times they are administered/day), tolerance, and the presence of antiviral resistance. Most HIV PIs are metabolized by the liver through enzymes that metabolize the drug cytochrome P450 (CYP450). Importantly, all approved HIV PIs have a high potential for drug–drug interactions due to their ability to inhibit drug metabolizing enzymes, most commonly cytochrome P450 3A4 (CYP 3A4) [10,11].

RTV is the strongest inhibitor of CYP 3A4 and, for this reason, is often combined in small doses (100 to 200 mg daily) with other PIs to produce a booster effect, increasing plasma levels and the half-life of the protease, without significantly increasing side effects [12,13].

COBI is a newly introduced pharmacological enhancer that has inhibitory activity against several drug metabolizing enzymes in addition to CYP 3A4 including CYP 2D6 and the P-glycoprotein (P-gp) transporter, making it an important agent. It is used in therapy when the increase in active substance levels of agents metabolized by the cytochrome P450 system is desired, being available as 150 mg tablets [14].

Ten HIV PIs are currently available: saquinavir, indinavir, RTV, nelfinavir, amprenavir, fosamprenavir, lopinavir, atazanavir, tipranavir, and DRV. PIs represent the largest class of drugs used in the fight against HIV. These substances block the activity of HIV protease, an enzyme that the virus uses to break down high-mass polyproteins into smaller units needed to assemble new viral particles [15].

PI acts in the late stage of the HIV replication process. When the virus infects a cell, its replication systems are forced to produce long viral precursor proteins. These precursors are grouped so that they are able to leave the cell, causing cell lysis and its death. Even before proteins leave the cell, they must be cleaved by an enzyme called “HIV protease” (a member of the aspartyl-protease family of enzymes, which includes renin and pepsin). The protease acts in the budding virion, causing the morphologically distinguishable immature virions to mature. At such a late stage in replication, the benefit of this action is the relatively small locus of activity. The requirement that all viral proteins be enzymatically cleaved at this location creates a blockage and involves a very specific and effective action of disrupting viral replication. However, there is a disadvantage of this mechanism, namely that these substances are not potentiated and acted upon at the initial time of infection. Thus, PIs have a virustatic rather than a virucidal effect [7].

However, HIV can still replicate (even in the presence of PIs), but the resulting virions are immature and do not have the ability to infect new cells. All molecules currently known to act by inhibiting HIV protease are metabolized by CYP450, especially by the CYP3A4 isoenzyme group.

If a CYP 3A4 inhibitor (RTV or COBI) is administered associated with both fluconazole (antimycotic, also an enzymatic inhibitor of CYP 3A4) and atazanavir (PI), the rate of metabolization of all ARVs decreases, leading to their increased concentration in plasma. The action of other drugs also metabolized by CYP 3A4 isoenzymes (i.e., rifampicin, sildenafil, tacrolimus) will be potentiated, increasing the risk of toxicity or, conversely, blocking the activity of other substances by hepatic metabolism. CYP3A4 inducers decrease the PI concentrations although this effect has been shown to counterbalance the effect of stimulators. When concentration problems occur, monitoring plasma concentrations (at the initial moment—T_0_, then at 1, 2, 3, and 4 h) may be helpful to clinicians in understanding whether the maximum concentration and prolonged exposure exceed the therapeutic safety limits.

Administered on a long-term basis, most of these PIs have numerous side effects, the most common being metabolic syndromes: dyslipidemia [16], insulin resistance [17], and lipodystrophy/lipoatrophy as well as cardiovascular [18] and cerebrovascular diseases [16,19,20,21,22]. Used in monotherapy, the PIs are associated with a slight improvement in the body fat distribution [23,24].

HIV PIs approved by the Food and Drug Administration (FDA) have some structural similarities and a similar binding pattern, which may be responsible for some of the common side effects of the regimens containing them.

## 3. Antiretroviral Therapy Resistance

ART promotes the development of resistant viral variants extremely fast, within a few days or weeks from the moment of incomplete suppression of replication [6]. The virus suffers mutations through the modification of a single nucleotide or the deletion of some nucleotides [25]. The accumulation phenomenon for certain resistance mutations in some cases may be connected with lower “viral fitness”, that is, lower viral replication capacity. HIV can generate variants that present stable infectious capacity in the environment in which they are found [26,27]. Rapid selection of viral species (resistant to currently used inhibitors) is determined by the following factors: high rate of mutations (caused by lack of correction activity of viral reverse transcriptase); dynamic viral replication in HIV-positive subjects; potential double infection; and insufficient drug efficacy. These mutations occur not only in the protease substrate binding site in the immediate proximity of an inhibitor, but also outside the active site of the enzyme. In addition to the common amino acid substitution mechanism, insertions in the HIV-1 (PR) protease (selected during ART) were also observed [28,29].

HIV-1 protease is prone to resistance development largely due to the process in which it is synthesized in vivo. Reverse transcriptase creates viral deoxyribonucleic acid (DNA) from viral ribonucleic acid (RNA) that is embedded in the virus host cell during infection. This process, unlike eukaryotic DNA synthesis, is highly prone to errors due to the lack of correction mechanisms, causing HIV-1 to develop many polymorphic mutations [30,31].

The high error rate of this process increases the probability that genetic mutants will be introduced into the system being influenced by the evolutionary pressures of PIs. Consequently, the resistance development is exacerbated and persistent due to the limited ability of many PIs to maintain effective concentrations in the body and/or to reach the desired sites of action such as the central nervous system (CNS) [32]. The CNS and other regions of the body can act as viral reservoirs, which can lead to increased virulence [33].

To improve the effective penetration of PIs, strategies have been used to increase the ability to cross the blood–brain barrier (BBB) by increasing the lipophilic nature of inhibitors and introducing fluoride into the structure in different positions [32,34].

### Enzymatic Resistance to Protease Inhibitors

Resistance to PIs occurs due to mutations in the HIV protease. Currently, about 10–20 such mutations are known. Frequently, multiple mutations can occur/exist in the same patient, which leads to the appearance of totally different enzymatic structures [35]. Published data showed that mutations in 45 of the 99 amino acid positions in protease are associated with PI treatment [36]. Mutagenesis can occur spontaneously because the reverse transcriptase present in the virus has no restorative properties, leading to a high rate of mutagenesis [37,38]. Some studies also point out that low adherence to antiviral pharmacotherapy promotes the selection of strains resistant to PIs [39,40]. Mutants that confer resistance to ARV can result mainly from two processes: (1) replacement of a single amino acid (from the 99 amino acid positions mentioned above, in the protease), which is the principal pathway; and (2) in some cases, an amino acid is inserted into the gene that encodes amino acid sequencing in the structure of the protease [29,41,42].

The evaluation of the structural changes and the activity of the mutant proteins highlighted three categories of mutations, as follows:

- Unique amino acid substitutions in the cavity of the active site of the enzyme, altering the size and shape of the side chains, which may alter the affinity of the drug and preserve the catalytic activity. The known mutations of this type are D30N, V32I, I47V, G48V, I50V, Val82, I84V, and G48T/L89M [43].
- Mutations that reduce the stability of the protease dimer, increasing dissociation between these portions and releasing limited inhibitor. The mutations associated with this mechanism are L24I, I50V, F53L, and Ile50 [44].
- Distal mutations, also related to PI resistance, may occur in different ways and may be difficult to detect, but may promote structural changes until the interaction with the inhibitor changes [37,45]. Distal mutations can also cooperate with active-site mutations to provide high PI resistance [46]. Examples of these mutations are L76 V [47,48] G73S [44], and N88D [49].

The highest resistance to PIs is correlated with primary and secondary mutations in the protease gene. Primary mutations are caused by changes in the active locus of the protease; secondary mutations help restore viral protease activity, increasing viral replication capacity. Typical examples are V82A/F/T mutations, which contribute to the resistance to many PIs including lopinavir/RTV, atazanavir, fosamprenavir, nelfinavir, and saquinavir. Cross-resistance to other ARVs has also been studied in people infected with HIV using more than 6000 clinical isolates. These studies indicated that 59–80% of isolates had reduced susceptibility to indinavir (IDV), RTV, nelfinavir (NFV), and saquinavir (SQV), among other PIs [50].

The most common and general approved PIs have lower than optimal pharmacokinetic properties. This is due to the fact that PIs are only substrates for the CYP3A4 isoenzyme (a subunit of the hepatic cytochrome P450 enzyme system, which is responsible for the metabolic degradation of PIs) [51].

As a result, numerous approved PIs have low plasma levels and short half-life. Interestingly, some PIs act as both inhibitors and inducers of isoenzymes. As it turns out, RTV is a potent inhibitor of CYP3A4 and the administration of a low dose of RTV with PI significantly improves the pharmacokinetic parameters of PIs. PIs such as DRV, fosamprenavir, and atazanavir can be given as a once daily dose when given with a low dose of RTV [52].

However, the use of RTV as a pharmacokinetic booster may raise some issues. When RTV is used as an enzyme inhibitor for other classes of antiviral compounds in HAART that does not contain PI, a subtherapeutic dose of RTV may accelerate the onset of PI-resistant HIV-1 variants [53]. Multi-resistant HIV-1 variants that are resistant to one PI are also likely to be cross-resistant to all PIs. RTV has other side effects such as lipid disorders and unwanted drug interactions. Recently, a new pharmacokinetic enhancer has been developed, COBI, which is stable and has no anti-HIV activity [54,55].

Unlike RTV, COBI will not contribute to the development of drug-resistant HIV-1 variants. The formulation of a PI with COBI resulted in an all-in-one pill, convenient for patients, resulting in increased adherence by reducing the pill burden. While this regimen has improved the pharmacokinetic properties of currently approved PIs, a PI design that does not require a pharmacokinetic booster is the ideal option.

## 4. Pharmacokinetics of Antiretrovirals (ARVs)

**Figure 1:**
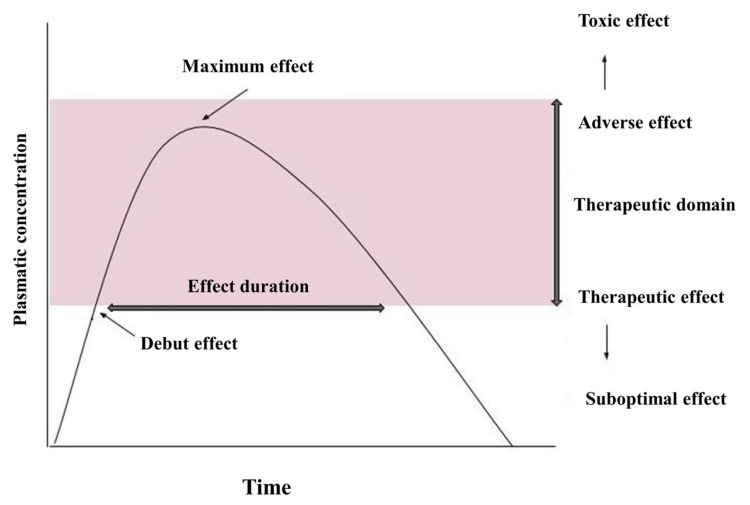
Plasmatic concentration–time curve.

Pharmacokinetics describes the quantitative aspects of the pathway of the drug substance in the body after administration [52]. Figure 1 shows a curve of plasma concentration over time after a single dose of drug. After oral administration, the drug enters the gastrointestinal tract, dissolves, and is absorbed through the intestinal wall. It then follows the hepatic pathway and enters the systemic circulation. At this time, it can be detected and quantified in plasma [53].

Absorption may take some time; the interval elapsed from the time of administration until the occurrence of a certain level of concentration in the blood is called “lag time”—the period of delay. Absorption determines the level of plasma concentration [53]. The stages of metabolism follow the distribution in the compartments of the body and elimination, in unmodified form, through the liver, kidneys, or both, being metabolized in various ways/organs, the most important being the liver. When the level of the medicinal substance entering the plasma by absorption is equal to the level of the substance that is distributed or eliminated, the maximum concentration level (Cmax) is reached. The time at which this concentration is reached is called Tmax. After this point, the distribution and elimination rates exceed the absorption rate, and the plasma concentration begins to decrease [52,53].

If the distribution takes place faster than elimination, a biphasic decline will be observed on the curve. The first curve that will decrease, much faster, results from the distribution of the drug in the compartments of the body. The second curve occurs later, is slower, and represents a decrease in the plasma concentration of the substance after elimination (being slightly counterbalanced by redistribution from tissues, again into plasma). The time when/at which the plasma concentration of the drug falls below 50% is called the half-life (t1/2). Due to the different distribution and elimination, there are two half-lives, but in practice, no distinction is made between them [53,55].

At the end of the dosing interval, before the ingestion of a new dose, a residual concentration (Ctrough) is reached, which is most often equal to the minimum concentration (Cmin). The area under the curve (AUC) is a pharmacokinetic parameter that measures drug exposure over the entire dosing interval, without providing information if these pharmacokinetics have a dynamic profile (i.e., if there is a large difference between the maximum concentration (Cmax) and the minimum concentration (Cmin)) [53].

In the case of ARV drugs, this link between active substance concentrations, efficacy, and toxicity levels has been demonstrated, therefore Cmax, Cmin, and AUC are significant parameters. They indicate the degree of ARV exposure, either at a certain point in time (Cmax and Cmin) or at a certain time interval. The pharmacokinetics of any drug change with a single dose or with repeated doses. When the pharmacokinetic profile does not change after the next dose, an equilibrium level is reached, called the steady state. The time to reach this threshold is different and depends on a number of factors. Among ARV drugs, those that are capable of inducing CYP 450 enzyme systems need 14 days to reach steady state plasma levels [56].

Another important factor in reaching the level of therapeutically effective plasma concentration is the binding to plasma proteins. The most important plasma proteins are albumin and alpha I-acid glycoprotein (AAG). AAG mainly binds HIV PIs and has a wide variety depending on race, body mass index (BMI) (stronger in those with high body mass index), and the stage of HIV infection (strong binding in the acute phase of the disease). Most ARV drugs are bound to plasma proteins, sometimes even completely. Only the free, unbound fraction is pharmacologically active, so the factors that prevent this binding influence the effectiveness of the therapeutic agent. The determination of the plasma drug concentration takes into account both the bound and the unbound fraction of plasma proteins [53].

It is known that pharmacodynamics studies explain and describe the response/concentration relationship. When this correlation exists, plasma levels can be manipulated by adjusting the dose in order to obtain concentration values within a desired necessary therapeutic window (the range of concentrations in which the toxicity/efficacy ratio is optimal). In practice, therapy monitoring can be used to correctly adjust the dose. If low doses are given for long periods of time and the level of plasma concentration falls below the optimum concentration, there is an increased risk of therapeutic failure due to the appearance of viral resistance and ART will be ineffective. On the other hand, too high doses of drug are associated with an increased risk of toxic effects, influencing the patient’s quality of life, with immediate consequences on their adherence to treatment [1].

The pharmacokinetics of ARV substances are extremely different, depending on the concentration but also on the patient, with multiple side effects and high toxicity. Clinical studies have shown that there is a great variety in terms of therapeutic response, from one patient to another or for the same patient, depending on many variables/circumstances [1,52,53,54,55,56].

### 4.1. Pharmacokinetics of Protease Inhibitors

Oral bioavailability of PIs is generally considered to be weak or variable (i.e., less than 68%). On average, the time to Cmax among agents is 3 h (range, 1, 5–6 h), with a median half-life of approximately 6 h. The elimination half-life varies between 3 and 15 h. Except for indinavir (Crixivan, IDV, Merck, Germany), all PI agents are extensively bound to proteins (>90%). Of all the pharmacokinetic parameters, the metabolic considerations on PIs may be the most worrying. This is especially true for patients receiving poly-medicine (i.e., antifungal or antibiotic agents for AIDS, associated with comorbidities and at risk for multiple drug interactions). PIs are inducers and substrates of multiple cytochrome P450 (CYP) isoenzymes including CYP 3A4, CYP 2D6, CYP 2C9, and β-glycoprotein.

Compared to other classes of ARVs, PIs have unique characteristics that justify their prescription compared to other classes of drugs [56].

#### 4.1.1. Intrinsic Antiviral Activity

A recent study pointed out that the intrinsic antiviral potency (called an instantaneous inhibitory potential) may be higher for PIs than for other classes of ARV drugs. This observation is based on the fact that nucleoside analogues and integrase inhibitors target reactions in which a single enzyme complex and viral nucleic acid mediate a key step. Instead, PIs target the entire number of enzymes [57].

#### 4.1.2. Residual Activity

Studies have found that PIs show residual ARV activity, even in individuals who have already developed resistance to other antiretrovirals. A significant proportion of newly developed viruses will remain partially sensitive to PIs for some time. This explains the need to continue a failed treatment regimen with PIs instead of eliminating them, in the case of patients who have no other treatment options. This is because the use of PIs is associated with low viral load and viremia below 10,000 copies/mL, which does not lead to a decrease in CD4, at least for 1–2 years [58,59].

#### 4.1.3. Increased CD4 Levels

Several studies comparing NNRTIs and PIs have shown that cluster gains of differentiation 4 (CD4) are generally higher using PIs than NNRTIs, even without dose adjustment in the case of patients undergoing complete viral suppression [60,61,62].

#### 4.1.4. The Unique Features of PIs Are as Follows:

- Reduced immune activation [63];
- Reduction of CD4+ cell apoptosis [64,65];
- Decreased fitness of PIs-resistant viral variants [66];
- Affected replication of PIs-resistant viruses in T-cells (tymocytes) [67].

From a clinical point of view, these characteristics make PIs a preferred class of ARVs, especially in patients with very low CD4 counts and whose priority is rapid immune restoration.

#### 4.1.5. Entering Sanctuaries

One of the main limitations of most PIs is the low penetration into the central nervous system (CNS). This feature may not be relevant in the case of ARV regimens in triple combinations [68].

#### 4.1.6. Genetic Barrier Against Resistance

The availability of PIs marked an important step in the treatment of HIV infection. However, there was a significant cost in terms of quality of life because first-generation PIs required complex dosing and had significant side effects. The latest PIs have overcome these limitations with a more convenient dosage (only a few pills, once a day) and a favorable safety profile and greater potency. The pharmacokinetic profile has improved considerably with low doses of RTV, and PIs continue to be key agents in the treatment of HIV [69]. The main enzymes responsible for the oxidative processes of exogenous and endogenous substances are CYP 450, they exist in more than 50 different isoenzymes in humans, metabolizing most foods and drugs (70–80%) [70]. Enzymes involved in the metabolism of ARV agents include CYP2D6, 2C9, 2C19, 1A2, 2EI, 2B6, and 2A6; however, many of them are subject to hepatic metabolism mediated by the CYP3A4, CYP3A5, and CYP2C9 isoenzymes [50].

To minimize the metabolism of these enzymes, pharmacokinetic enhancers (PK enhancers) like RTV and COBI are used as ART due to their ability to inhibit CYP3A4, CYP3A5, and CYP2C9; this reduces the dose and frequency of PI administration [71,72]. Monitoring the drug therapy in HIV-infected patients treated with DRV (PI) in combination with RTV (booster) or in combination with COBI (booster) provides clinicians with a new approach to the pharmacokinetics of these drugs.

### 4.2. Efficiency of PIs, Compliance, and Survival

People living with HIV must follow strict treatment regimens to prolong their lives and reduce mortality [73]. Lack of adherence and compliance leads to continued replication and the emergence of HIV resistance to ART. This viral resistance leads to aggravation of the patient’s condition, therefore, obtaining patient adherence is a major goal of therapy [15]. There are several causes that contribute to non-adherence, the main one being the occurrence of significant side effects or secondary mortality [2,74,75]. For example, in many HIV-infected patients who were given ARV drugs, liver failure was observed, and major complications were reported [76,77,78]. Although opportunistic infections are another important risk in the treatment of HIV+ patients, the real cause of death is most often an organ failure due to the adverse effects of ARV [79]. ARVs can cause damage to the gastrointestinal and liver cells, leading to lipid imbalance and sexual dysfunction [80,81,82]. As numerous studies show, all the above-mentioned side effects cause the patient to give up treatment [1,83,84].

### 4.3. PI Side Effects

The most common side effects reported by patients treated with PIs are as follows: sexual dysfunction, gastrointestinal disorders (nausea, vomiting, diarrhea), liver disorders, pancreatitis, cardiovascular disease, rash, and metabolic disorders. ARV intolerance and toxicity are the cause of frequent changes in treatment regimens. Unfortunately, this change also leads to the emergence of resistance [85,86,87,88,89].

Progress in treatment and individualization of doses has led to fewer side effects compared to the initial regimens. Today, less than 10% of naive patients (those who are in the first treatment regimen) on ART still experience adverse events that could limit the treatment. Even so, long-term complications persist, especially the appearance of insulin resistance and disorders related to lipid metabolism. Therefore, it is necessary to continue monitoring the complete blood count and metabolic profile of these patients. Tests should be performed every three to six months, being also necessary an annual analysis of the urine (in order to verify proteinuria) and a lipid profile; if the treatment schemes are changed, the tests must be done again, respecting the periodicity [5,89,90].

#### 4.3.1. Insulin Resistance

Insulin resistance is a common subject in HIV patients, contributing to dyslipidemia and impaired glucose metabolism [91]. Immune system activation results in chronic inflammation (high levels of proinflammatory cytokines) that may be prominent in patients undergoing treatment or may be improved in untreated patients [61]. Treatment with ARVs decreases proinflammatory cytokines, but insulin status still exists due to factors such as virus production, deterioration of lymphoid structure, and loss of Sterol-Regulatory Element-Binding Protein 1 (SREBP-1), which regulates the expression of genes associated with lipid synthesis. In addition, it is possible to induce endoplasmic reticulum stress and reduce glucose transporter 4 (GLUT4). Reduction of GLUT4 may lead to insulin resistance; approximately 80% of patients receiving these drugs develop insulin resistance, which may lead to diabetes [92,93]. PIs have different reactions in relation to insulin resistance; this difference in each drug behavior can be explained by distinct molecular targets for each PI or by the difference in affinity between molecules, even in the case of the same target cellular protein [21].

#### 4.3.2. Associated Metabolic Disorders

Metabolic disorders associated with PI therapy are the most problematic adverse events that may occur, their incidence varying from drug to drug. These disorders include hyperlipidemia, abnormal fat distribution (lipodystrophy), and impaired glucose tolerance, which are thought to be due to peripheral insulin resistance. Clinicians caring for patients treated with PIs should be alert to adverse events, especially those involving changes in lipid and glucose metabolism [94]. Lipid disorders are related to high levels of total cholesterol, low-density lipoproteins, very low-density lipoproteins, triglycerides, and an atherogenic lipid profile. Up to half of patients may have lipodystrophy syndrome (disorders and morphological changes related to lipid abnormalities) [95]. The first changes usually appear a few weeks after starting treatment.

Two studies published in 2007 on 25,000 patients showed that the use of PIs is directly correlated with myocardial infarction [96,97]. The first generation of PIs interferes with extracellular regulatory kinase (ERK) 1/2, GLUT4, and Akt/protein kinase B, resulting in effects on lipogenesis. Newly developed compounds such as atazanavir or DRV have fewer side effects of this kind [98].

#### 4.3.3. Lipodystrophy

Lipodystrophy is the redistribution of fat, with accumulation on the throat and abdomen, occasionally forming a “buffalo hump” (dorsocervical fat), being usually associated with fat loss from the face, buttocks, and extremities. This disorder is resistant to treatment, often not reducing even with the discontinuation of the triggering agent. Some patients become unrecognizable after the treatment because of the body and facial feature alterations, many of them resorting to plastic surgery for some relief. Glucose intolerance, which sometimes leads to diabetes and genuine diabetic ketoacidosis, have been reported more and more frequently [99].

Hyperlipidemia is closely associated with both hyperglycemia and redistribution of fat and may put HIV patients receiving HAART at increased risk for cardiovascular disease [100]. PIs suppress the breakdown of the nuclear sterol regulatory element-binding proteins (nSREBP) into adipose tissue. Suppression of nSREBP decomposition results in increased fatty acid and cholesterol biosynthesis in the liver, leading to lipodystrophy and insulin resistance in adipose tissue [101]. The combination of enzymatic inhibitors with lipid-lowering agents/“statins” may reduce lipid levels, possibly reducing the cardiovascular risk, but leading to rhabdomyolysis if the chosen statin is incompatible with this type of inhibitor [101].

Many patients experience gastrointestinal symptoms at the beginning of treatment with PIs or when switching from one regimen to another. These symptoms frequently lead to discontinuation of therapy and can be a major obstacle to efficient treatment [102]. The study found that 25% of patients discontinued treatment due to their toxicity regimens. Nausea and vomiting are among the most common complaints, up to 75% as presented in some studies [103]. These symptoms usually subside within 4–6 weeks of treatment, but some patients continue to experience long-term symptoms.

#### 4.3.4. Sexual Dysfunction

Sexual dysfunctions occur more frequently in patients treated with PIs than in patients treated with other substances [104]. There are studies showing that the rate of sexual dysfunction is 70% in the case of PI treatment [105]. However, there are no studies to explain the mechanism of erectile dysfunction, delayed ejaculation, and decreased libido as a result of PI therapy.

#### 4.3.5. Hepatic Toxicity

All drugs in the class of PIs have been associated with liver damage. About a third of patients treated with PIs have liver problems associated with high levels of enzymes. Most of the time, these reactions are mild and transient [106]. PIs are associated with four main forms of hepatotoxicity:

- Mild to moderate increases in serum aminotransferase and alkaline phosphatase levels that occur in a large proportion of patients taking ARV regimens containing PI. Moderate to severe increases in serum aminotransferase levels (above five times the upper normal limit) are found in 2% to 18% of patients, depending on the agent, the frequency of monitoring, and most importantly, the presence of virus coinfection with hepatitis B (HBV) or C (HCV). In HIV patients, without HCV or HBV infection (“mono infection”), increases in alanyl aminotransferase (ALT) and aspartate aminotransferase (AST) are uncommon; levels of more than five times the upper normal limit were reported in only 1–4% of patients. These increases are usually asymptomatic and self-limiting and can be resolved even with continued medication. Therefore, this should not be a reason to discontinue the medication.
- Hyperbilirubinemia, without other manifestations of liver damage, is caused by the inhibition of glucuronyl transferase UDP, the liver enzyme responsible for conjugating bilirubin that is deficient in Gilbert’s syndrome. Hyperbilirubinemia is usually mild, averaging 0.9–1.5 mg/dL, but may be more severe in patients with Gilbert’s syndrome with increases of 2.5 mg/dL or more and clinical jaundice. Jaundice, however, does not indicate liver damage. However, jaundice caused by PIs can be painful for the patient and is a reason to discontinue treatment.
- Acute, idiosyncratic liver damage has been reported in most agents not very frequently. The few reported cases usually occurred within one to 12 weeks after starting therapy, and the pattern of serum enzyme growth ranged from hepatocellular to mixed and cholestatic. Signs of allergy or hypersensitivity (fever, rash, eosinophilia) may also occur as well as the appearance of autoantibodies. Acute liver damage from PIs is usually self-limiting but severe and isolated cases of acute liver failure have been reported, especially in patients with pre-existing underlying liver disease.
- Exacerbation of chronic underlying hepatitis B or C in co-infected individuals. Hepatitis usually occurs in 2–12 months after starting therapy, being associated with a hepatocellular pattern of serum enzyme growth, and also by increases, followed by decreases in the serum levels of HBV DNA or HCV RNA. These fluctuations can be severe, and even fatal cases have been reported [14].

## 5. Drug–Drug Interactions

Drug interactions are the cause of many of HAART complications. Among them, an important role is played by the inhibition, respectively the enzymatic induction produced by PIs as well as the interactions of the P-glycoprotein.

### 5.1. Enzymatic Inhibition

Enzymatic inhibition occurs when a substance blocks or reduces the activity of metabolic enzymes. The net effect is to increase plasma levels and the effects of the parent compound, while preventing the formation of metabolites and reducing elimination. All PIs are inhibitors of cytochrome 450, especially in the CYP 3A4 isomorph. From this point of view, PIs behave similarly to ciprofloxacin, erythromycin, clarithromycin, diltiazem, itraconazole, or ketoconazole. They all inhibit CYP 3A4 and affect the pharmacokinetics of low therapeutic index substances. For example, in patients treated with carbamazepine, vomiting and dizziness, increased liver enzyme levels, and increased serum anticonvulsant levels were observed 12 h after a dose of RTV [107]. Even other PIs, although with lower action on CYP 3A4, affect the metabolism of some drugs such as HMG-reductase inhibitors, statins. The effect in this case is the more frequent occurrence of rhabdomyolysis [108].

The metabolism of the PIs is, in turn, affected by other potent 3A4 inhibitors, because all PIs are metabolized by the enzyme 3A4. Any substance with a stronger inhibitory effect on 3A4 can slow down the metabolism of PIs. The other potent inhibitors that influence the rest of the PIs include ciprofloxacin, clarithromycin, diltiazem, erythromycin, itraconazole, nefazodone, and grapefruit juice. Inhibition of PI metabolism by these agents may increase their efficacy or reduce the dose required (as seen when RTV is used to potentiate other PI). However, there is also an adverse reaction such as worsening of side effects and toxicity, which leads to an increased risk of events such as headaches, nausea, diarrhea, hepatitis, and pancreatitis. All lead, in the end, to non-adherence. It should be mentioned that the interruption of the potent inhibitor, other than PIs, entails a return to the uninhibited state, but also a rapid reduction in circulating levels of PI, which puts the patient in danger of developing viral resistance to the entire class of PIs.

### 5.2. Enzymatic Induction

This represents the phenomenon of increase or acceleration of metabolic enzyme activity, under the action of a substance or a compound. The net effect is to decrease the levels of the parent compound and its effects, while increasing the production of metabolites and the amount available for elimination. Rifampicin, carbamazepine, phenytoin, ethanol, and barbiturates are potent inducers of many enzymes belonging to the CYP 450 family [109]. Induction of PI metabolism can reduce their circulatory level, putting the patient at risk of developing viral resistance, which leads to failure of HIV treatment. P-glycoproteins can be inhibited and induced. They are found in many sites including the peaks of villi in the jejunal enterocytes and the endothelium of blood–brain barrier cells [110].

These glycoproteins are responsible for the outflow or “pumping” of substances outside the cells and are the “guardians” that influence the bioavailability and pharmacokinetics of drugs in the intestine and liver and prevent the penetration of the drug into the brain, testicles, and placenta. PIs are all substrates of P-glycoproteins, which may explain why it is difficult for ARVs to cross the blood–brain barrier or other sanctuaries. When P-glycoproteins are inhibited, the concentration of the drug increases, especially if it is a substrate for the P-glycoprotein. RTV initially inhibits the P-glycoprotein, which may allow other substrates to reach higher serum levels, either in the intestinal wall or in the blood–brain barrier. This inhibition of P-glycoprotein, together with 3A4 inhibition, would have an additive inhibitory effect and would increase the level of substrate drugs for P-glycoprotein and CYP 3A4. In vitro, long-term RTV exposure induces P-glycoprotein expression, reducing the drug ability to ‘cross the barrier’, and effectively reducing the bioavailability of P-glycoprotein as a substrate [7,111].

## 6. Protease Inhibitors Enhancement

ART combined with two or more PIs has changed the standard of care in the treatment of HIV infection. Two PIs regimens such as DRV/RTV are commonly used as initial therapy with PIs. As viral resistance increases and the development of new PIs decreases, clinicians resort to dual RTV-boosted PIs to treat patients. The potency of these combination therapies is increased, while the burden of pills, dietary restrictions, and often side effects are reduced. These clinical advantages lie in the improvement of the pharmacological properties of the substances, in particular, through changes in pharmacokinetics (absorption and metabolism). Changes are occurring in the absorption and metabolism of PIs when co-administered with a cytochrome P450 enzyme inhibitor (CYP) (e.g., a low dose of RTV is reflected in significant changes in pharmacokinetic parameters). The ability of RTV to increase concomitant plasma concentrations (Cmin) of co-administered PI is probably the greatest clinical benefit of dual therapy, as inadequate ARV concentrations may support long-term ARV resistance.

The fact that there is a close link between the therapeutic response and the plasma concentration of the drug substance is certain. Studies show that inadequate levels of circulating substance play an important role in therapeutic failure and the emergence of viral resistance [10]. Low adherence, low absorption, and high variability of pharmacokinetic parameters (in the case of schemes containing a PI) lead to poor exposure of the body to the drug substance and, consequently, to therapeutic failure. The combination of two or more PIs has favorable effects on the plasma levels of the substance available for action, increasing the PI potency by increasing Cmin [112]. At the same time, combined therapies increase tolerability by maintaining the plasma concentration in the effective area, without fluctuations exceeding the Cmax value, which leads to the occurrence of toxicity phenomena associated with medication. To these, the patient’s compliance with the dosage (by reducing the number of daily administrations) must be added. Food restrictions are also reduced. The first inhibitor used to enhance the effect of PIs was RTV, in a low dose of 100–200 mg [71], acting at the intestinal and hepatic level. Despite the immediate advantage over PI pharmacokinetics, RTV also has disadvantages. Having its own antiviral activity, the first problem that arises is that of the resistance that can be installed. To this is added the poor solubility of the substance, which limits co-formulation with other ARV agents [113].

RTV also has tolerability problems in a large number of patients. Other disadvantages of RTV are that it inhibits/induces other enzymes that metabolize drugs, resulting in a significant number of drug-drug interactions [113]. Based on these data, other boosting agents were formulated. This is how COBI, a structural analogue of RTV, appeared without antiviral activity and with improved physicochemical properties [53,114].

COBI’s CYP 3A4 inhibitory potency is similar to that of RTV [115]. A total of 150 mg (unique dose) of COBI daily provides bioequivalent exposures compared to 100 mg daily of RTV as follows: Atazanavir, 300 mg once daily [116]; DRV, 800 mg once daily [117]; and Elvitegravir, integrase inhibitor, 150 mg once daily [118].

COBI is marketed as a single agent or in fixed combinations, in association with atazanavir, DRV, or elvitegarvir. The two boosters, COBI 150 mg/day and RTV 100 mg/day, may be interchangeable with similar therapeutic results. However, clinicians prefer the administration of 150 mg COBI as a boosting agent for CYP 3A4 metabolites rather than RTV due to its higher specificity and the fact that it has no enzyme induction effect. Hence, the profile of interaction with other drugs is different.

## 7. Pharmacokinetic Enhancers: Cobicistat (COBI) or Ritonavir (RTV)

Although both COBI and RTV have similar boosting profiles for CYP3A4 and can be used with the same therapeutic efficacy, COBI is preferred due to its greater specificity and the absence of inducing properties. In addition, the two substances interact differently with other biochemical/medicinal substances metabolized by the following enzymes: CYP1A2, CYP2B6, CYP2C8, and CYP2C9. In this context, one of the biggest challenges for clinicians is to adjust doses in patients with multi-drug therapy:

- RTV, marketed as Norvir; it is included in Kaletra—lopinavir/RTV;
- COBI, commercially called Ybost; it comes in many combinations: atazanavir/COBI (Evotaz), elvitegravir/COBI/emtricitabine/tenofovir (Genvoya), DRV/COBI (Resolve), elvitegravir/covetousness/emtricitabine/tenofovir disoproxil (Stribild), DRV/COBI/emtricitabine (Symtuza).

### 7.1. Ritonavir

Ritonavir (RTV), C_37_H_48_N_6_O_5_S_2_, is an ARV drug in the class of systemic PIs used in combination with other drugs for the treatment of HIV/AIDS. It was approved for commercialization on 1 March 1996, becoming the seventh ARV and the second PI entering the U.S. market. This class of ARVs has played an extremely important role in reducing the number of deaths caused by HIV/AIDS. The introduction of HAART at that time in the United States led to a decrease in the annual death rate from 50,000 to 18,000 [119]. RTV is an L-valine, L-valinamide derivative in which the alpha-amino group has been acylated by a ((2-isopropyl-1,3-thiazol-4-yl) methyl) methyl carbamoyl group and in which a hydrogen amino-carboxamide was replaced with (2R, 4S, 5S) -4-hydroxy-1,6-diphenyl-5—((1,3-thiazol-5-ylmethoxy) carbonyl) amino- hexane-2-yl. The molecular weight is 720.95 (g/mole), it has a poor solubility, and a terminal half-life of 3–5 h. Plasma protein binding is done in a proportion of over 98%, passing into the cerebrospinal fluid (CSF)-to-plasma ratio being 0.7% [12].

**Figure 2:**
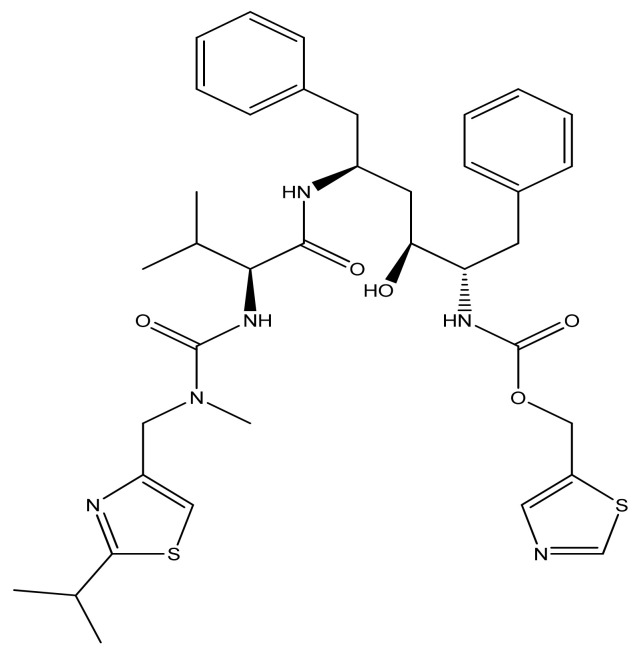
Chemical structure of ritonavir (RTV).

The chemical structure of RTV is shown in Figure 2.

#### 7.1.1. Mechanism of Action

RTV is a pseudo-C2-symmetric small molecule inhibitor [120]. It has a high affinity for several cytochrome P450 isoenzymes (CYP) and may inhibit oxidation in different degree of magnitude (CYP3A4 > CYP2D6) as well as inhibiting CYP2C19, CYP2C8, and CYP2C9 [121,122]. Additionally, it manifests enzymatic induction properties on CYP1A2, CYP2C19, CYP2C8, CYP2C9, CYP2B6, and UGT1A4 [123]. Co-administration of RTV and medicinal products may increase the plasma concentrations of these medicinal products, leading to an increase or prolongation of the therapeutic effects and the frequency of adverse reactions [124,125].

RTV inhibits the P-glycoprotein (P-gp) and the cellular transport mechanism via this efflux pump. Inhibition of P-gp may contribute to the stimulation of drug effects by interrupting the active transport of PI in the cells of the intestinal tract, liver, and kidneys. The inhibitory effect of RTV (with/without other PIs) on P-gp activity may decrease over time. RTV may induce glucuronidation and oxidation by CYP1A2, CYP2C8, CYP2C9, and CYP2C19. Thereby, RTV may increase the biotransformation of drugs metabolized through these pathways and may decrease systemic exposure to these drugs whose therapeutic effect may decrease or may be shorter. The effect of enzyme modulation may be dose dependent [121].

#### 7.1.2. Pharmaceutical Form and Therapeutic Indications

RTV is available as an oral solution (for children) and as a film-coated tablet containing 100 mg of the active substance. It is used in combination with other ARVs to treat HIV-1 infected patients, adults and children over two years of age. As a pharmacokinetic enhancer, in combination with DRV 600 mg, RTV 100 mg is administered twice daily to patients previously treated with ARV. For those not previously treated with ARV, DRV 800 mg and RTV 100 mg are given once a day [126].

#### 7.1.3. Pharmacodynamic Properties

**Figure 3:**
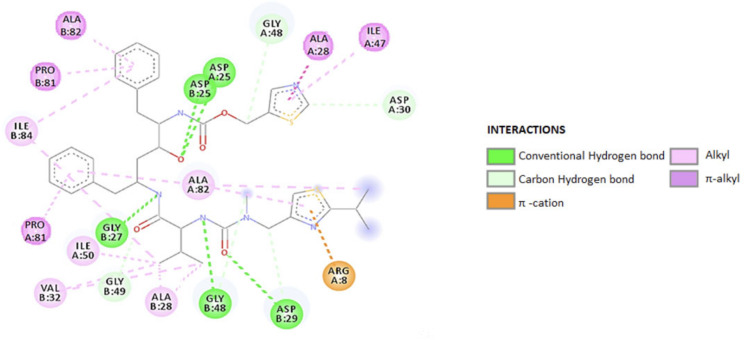
2D diagram representing the links between ligand and protein. (Discovery Studio Client version 19. 1.0. 18287; Biovia Discovery Studio Visualizer, Dassault Systèmes Corporate, Waltham, MA, USA; accessed on 28 December 2020). Legend: glycine (GLY27, GLY48, GLY49); alanine (ALA28, ALA82); arginine (ARG8); aspartic acid (ASP25, ASP29); proline (PRO81); valine (VAL32); isoleucine (ILE47, ILE50, ILE84); A and B are the chains.

**Figure 4:**
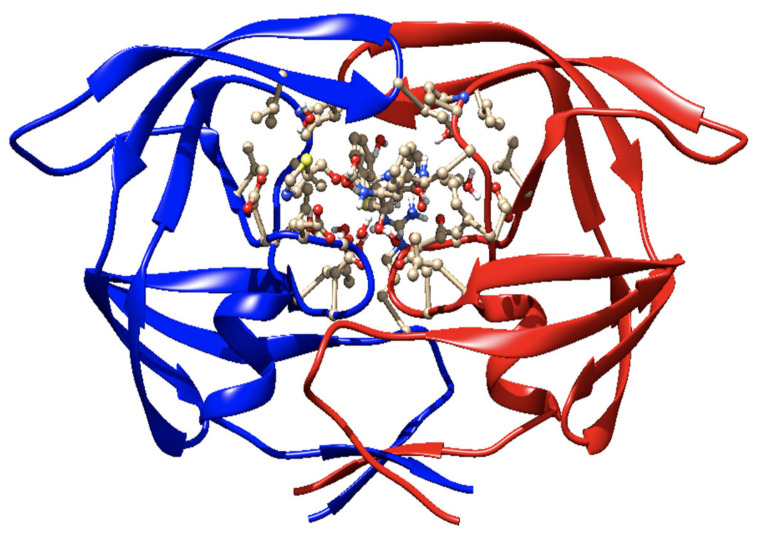
3D structure of Ritonavir-HIV-1 protease complex (UCSF Chimera version 1.14 (build 42091), accessed at 28 December 2020, San Francisco, California, USA). Legend: Blue – chain A, Red – chain B.

(a) RTV as a pharmacokinetic enhancer. The pharmacokinetic potentiating effect of RTV is based on its potent inhibition action on CYP3A-mediated metabolism. The degree of enhancement is related to the metabolic pathway of the co-administered PI, and to the impact of that PI on RTV metabolism. Maximum metabolism inhibition of the co-administered PI is generally achieved with RTV doses between 100–200 mg, twice daily [72].

(b) RTV as an ARV. It is an orally active peptidomimetic inhibitor of HIV-1 and HIV-2 aspartyl proteases. Inhibition of the HIV protease renders the enzyme unable to process the gag-pol polyprotein precursor, leading to the production of HIV particles with immature morphology that are unable to initiate new cycles of infection. RTV has selective affinity for HIV protease and has low inhibitory activity on human aspartyl proteases. RTV binding to HIV protease is shown in the docking patterns in 2D (Figure 3) and 3D (Figure 4) [127]. The molecular docking was done using the crystal structure of wild type HIV-1 protease, expression system Escherichia coli BL21 (DE3). Expression systems are genetic constructs (a gene encoded by deoxyribonucleic acid (DNA)) that are designed to produce a protein, or a ribonucleic acid (RNA), either inside or outside a cell, being used in research and in the commercial production of enzymes or therapeutics.

From the analysis of the 2D diagram, it is revealed that RTV binds to the following amino acids of the wild type HIV-1 protease: GLY27, ALA28, VAL32, ASP29, ALA82, ALA28, PRO81, ARG8, and ILE47. ASP25 forms hydrogen (H) bonds to the oxygen in the HIV protein side chain, ALA28 and PRO81 form π–alkyl bonds via chains A and B. The amino acids ALA28 and ILE50, in the HIV-1 chain A and B establish π–alkyl and alkyl bonds with RTV. ILE47 and ALA28 form a π–alkyl bond with the 1,3-thiazole group of the ligand, ASP30 through C–H, and ALA82 through both the C–H and the π–alkyl bonds. ARG8 bonds via π–cationic with the protein.

In RTV docking with the B-chain of HIV-1, the main link is π–alkyl. PRO81 is formed by the pyrolidine protein bond through the benzene ring. The amino acid ASN25 in the A chain as well as in the B chain (through the C–H and π–alkyl) bonds and interacts at the same oxygen atom in the ligand structure.

ILE84 forms a π–alkyl bond with benzene and C-alkyl in the ligand side chain. VAL32 binds by alkyl, and GLY27 and ASP25 by the H bond. ASP29 and GLY49 to O, in the side chain, form the C–H bond, and ASP29 also forms the H bond. Table 1 summarizes the ligand–receptor interactions, HIV-RTV protease, at the A and B chains of the protease, the distance between them and the types of connections that occur.

#### 7.1.4. Pharmacokinetic Properties

Absorption. According to the summary of product characteristics (SPC), a study of HIV-infected volunteers evaluated the pharmacokinetics of RTV multiple-dose regimens during fasting. RTV accumulation was observed to be slightly lower than that calculated on a single dose basis due to an increase in apparent dose-dependent clearance. Over time, the plasma concentration of RTV before the next dose decreases, possibly due to enzyme induction, but appears to stabilize after two weeks of treatment. The time to peak plasma concentration (Tmax) remained constant—approximately 4 h as the dose increased. Renal clearance was on average less than 0.1 L/h and relatively constant over the entire dosing range. It has been observed that foods slightly decrease the bioavailability of RTV film-coated tablets, especially foods with moderate and high lipid content [113].

Distribution. The apparent volume of distribution (VB/F) of RTV is approximately 20–40 L after a single 600 mg dose. In humans, RTV has been shown to be 98–99% bound to plasma proteins and is consistent at concentrations between 1.0–100 µg/mL. RTV binds with comparable affinities to both alpha-1 acid glycoprotein (GAA) and human plasma albumin (APU). Studies in rats have shown that the highest concentrations of RTV are achieved in the liver, adrenal glands, pancreas, kidneys, and thyroid. The tissue/plasma ratio was approximately 1 in the lymph nodes in rats, indicating that RTV is distributed in the lymph tissue. It slightly penetrates into the brain [125].

Drug metabolism. RTV is extensively metabolized by hepatic cytochrome P450, mainly by CYP 3A4 isoenzymes and, to a lesser extent, by CYP 2D6 isoforms. Animal and in vitro studies with human liver microsomes have shown that RTV is oxidatively metabolized. In humans, four metabolites of RTV have been identified. The major metabolite is isopropyl-thiazole (M-2), with antiviral properties similar to the parent compound. However, the AUC of metabolite M-2 was approximately 3% of that of RTV [128].

Clearance. RTV is eliminated primarily by the hepatobiliary route. Approximately 86% of the radiolabeled molecules were found in feces, some of which is thought to be unabsorbed RTV. In studies, the renal route has not been shown to be a major route of elimination of ritonavir [113].

#### 7.1.5. Pharmaco-Toxicology

The RTV target organs are the liver, retina, thyroid gland, and kidneys. Hepatic changes involved hepatocellular, biliary, and phagocytic structures and were accompanied by increases in liver enzymes. Human clinical trials did not reveal RTV-induced ocular changes. All thyroid changes were reversible upon discontinuation of RTV. Clinical investigations did not show significant impairment of the thyroid function tests or significant renal impairment in humans. There were observed various side effects: common (at low doses): diarrhea and increased lipid levels; and rare: changes in heart rate, severe allergies (hypersensitivity reactions) and eczema (Steven-Johnson syndrome) [14,72,129].

### 7.2. Cobicistat

Cobicistat (C_40_H_53_N_7_O_5_S_2_) is a structural analogue of RTV, in which the valine fragment was replaced with a 2-morpholinethyl group and the hydroxyl group was removed. These changes cancel out the anti-HIV activity per se of COBI, retaining only the highly inhibitory and selective effects on the CYP 3A protein isoenzyme family [120]. This way, the substance is able to increase the plasma concentration of other ARVs, when co-administered, without the risk of genetic mutations in HIV, resistant to COBI. It was synthesized by structure–activity relationship studies from RTV and deoxy-RTV. COBI is used in therapy for its ability to inhibit liver enzymes that metabolize other anti-HIV drugs, especially prosthetic and integrase inhibitors.

As a monocarboxylic acid amide obtained by the formal condensation of the carboxy group of (2S)-2-(((2-isopropyl-1,3-thiazol-4-yl) methyl) (methyl) carbamoyl- amino)-4-(morpholin-4-yl) butanoic acid with the amino group of 1,3-thiazol-5-ylmethyl ((2R, 5R)-5-amino-1,6-diphenylhexane-2-yl) carbamate, COBI has a molecular weight of 776 (g/mole), good solubility, and terminal half-life of 3–4 h. Plasma protein binding is 97–98% and CSF to plasma ratio is 1.1% [130].

**Figure 5:**
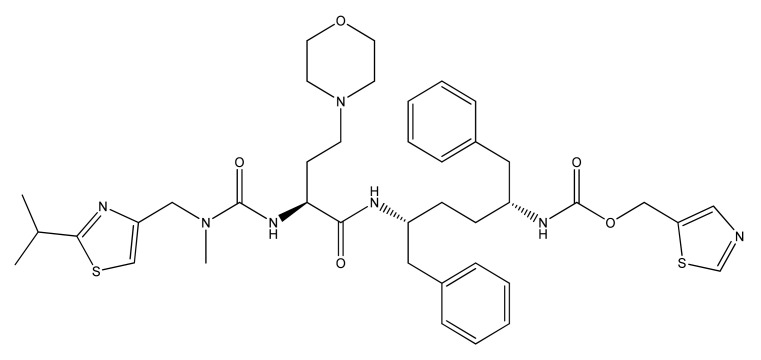
Chemical structure of cobicistat.

It acts as a pharmacokinetic enhancer in the treatment of HIV-1 by inhibiting the CYP P450 enzymes responsible for the metabolism of other drugs. It works as a CYP450 inhibitor. It belongs to the class of 1,3-thiazoles, but also to the class of morpholines, urea, is a carbamate ester and an amide of monocarboxylic acid. The chemical structure of the substance is shown in Figure 5.

#### 7.2.1. Mechanism of Action and Therapeutic Effects

Selective inhibitor of cytochrome P450 (subfamily CYP3A enzymes) with RTV-like potency, and COBI inhibits the mechanism of action of these enzymes. Inhibition of CYP3A-mediated metabolism enhances systemic exposure to drugs that are substrates for CYP3A (atazanavir or DRV), which have limited bioavailability after oral administration and a reduced half-life due to CYP3A-mediated metabolism. The inhibitory effect of COBI is also manifested on other enzymes: CYP 3A4, CYP2D6, P-gp, OATP1, MATE1, and BCRP [122]. COBI does not show an enzyme-inducing role [123].

#### 7.2.2. Pharmaceutical Form and Therapeutic Indications

Provided in the form of a film-coated tablet, COBI assures 150 mg active substance concentration. It is indicated as a pharmacokinetic enhancer for atazanavir 300 mg once daily, or DRV 800 mg once daily, as one of the components of ART combination in HIV-1 infected adults. It is recommended to be co-administered orally, both food and the drug. The tablet should not be chewed or crushed. COBI is formulated in fixed doses with atazanavir, DRV, and tenofovir/emtricitabine/elvitegravir [131].

#### 7.2.3. Pharmacokinetic Properties

Absorption. After oral administration of COBI with food to HIV-1 infected subjects, peak, plasma concentrations were reached 4 h post-dose. Mean steady-state Cmax, AUC, and Cper-dose values (mean value ± SD) after administration of multiple doses of COBI to HIV-1 infected subjects (*n* = 68) were 1.2 ± 0.3 µg/mL, 10.9 ± 0.8 µg h/mL, and 0.07 ± 0.07 µg/mL, respectively [132].

Distribution. COBI is 97–98% bound to human plasma proteins, and the ratio of mean plasma and blood drug concentrations was 2 [133].

Drug metabolism. It is metabolized by oxidation mediated (major pathway) by CYP3A and (minor pathway) by CYP2D6 and is not subject to glucuronidation. After oral administration, 99% of the circulating radioactivity detected in plasma was due to unmodified COBI. Concentrates of metabolites are low in urine and feces and do not contribute to the CYP3A inhibitory activity of COBI [133].

Clearance. After oral administration, 86% and 8.2% of the dose were found in feces and urine, respectively. The median elimination half-life of COBI after administration of Tybost (trade name) is approximately 3–4 h [53,134].

Pharmacotoxicology. Conventional studies on repeated dose toxicity, genotoxicity, and toxicity to reproduction and development have shown no risk to humans.

Side effects. As common side effects can be highlighted the following: high blood sugar, increased appetite, insomnia, drowsiness, nightmares, headache, dizziness, altered taste, nausea, diarrhea, abdominal pain, bloating, flatulence, dry mouth, jaundice, eczema and itch; rare side effects include kidney stones [135,136].

### 7.3. Similarities and Differences Between RTV and COBI

Table 2 presents the similar and different actions of the two drugs (COBI and RTV) [53,116,120,137,138,139,140,141,142,143,144,145,146].

## 8. Protease Inhibitor—Darunavir

**Figure 6:**
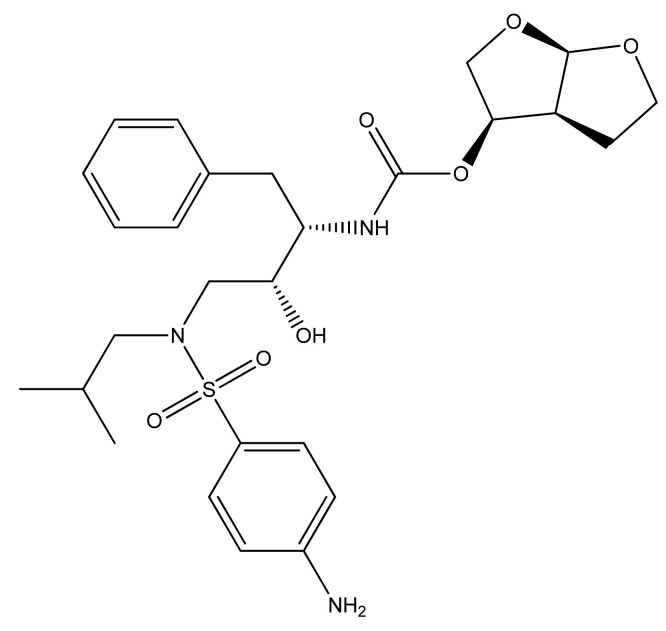
Chemical structure of darunavir.

DRV is the latest formulated PI [6] providing a high genetic barrier to the onset of viral resistance as well as effective antiviral activity on HIV-1 strains already resistant to other PIs [11]. 3-((4-Amino-benzenesulfonyl)-isobutyl-amino)-1-benzyl-2-hydroxypropyl)-carbamic acid hexa-hydrofuro-(2,3-b) furan-3-yl ester is a synthetic nonpeptide PI, from the class of systemic antivirals, developed in 1988. The molecular weight is 547.7 (g/mole), solubility of 0.15 mg/mL, and 95% plasma protein binding. The chemical structure of the substance is shown in Figure 6.

The compound was licensed for marketing in the United States of America in 2006 and in 2007 in Europe. Since 2016, DRV is a treatment option for children over three years of age, adolescents, and naive adults, infected with HIV-1, but also for critical patients [147]. Since its first administration, numerous studies have demonstrated the effectiveness of DRV against HIV-1, and this ARV drug has become an important component of HAART. It has a high affinity for HIV-1 protease, binds strongly to it by forming a stable complex due to flexible conformation, and skeletal interactions [122].

Low doses of RTV or COBI are used to increase DRV plasma level. It can also be used in prophylaxis. The administration is done once or twice a day, only according to a schedule established by the specialist. This regimen may include 800 mg of DRV and 150 mg of COBI, 800 mg of DRV and 100 mg of RTV (once daily). When given twice a day, 600 mg of DRV in combination with 150 mg COBI or 100 mg RTV should be taken [54].

### 8.1. Pharmaceutical Form

DRV is marketed as a 100 mg/mL oral suspension or as 75, 150, 300, or 600 mg film-coated tablets. In fixed doses, it is formulated with COBI (800 mg DRV/150 mg COBI). As with other ARVs, DRV does not cure HIV/AIDS [122].

### 8.2. Mechanism of Action

DRV is an inhibitor of dimerization and catalytic activity of HIV-1 protease, thus inhibiting proteolytic activity and subsequent replication of HIV-1. It also selectively inhibits the cleavage of Gag-Pol-encoded HIV polyproteins in virus-infected cells, preventing the formation of infectious mature viral particles [148].

In therapy, a low dose of RTV or COBI, in combination or not with other ARV medicines, should be used to treat patients infected with HIV-1, adult, adolescent, and pediatric patients over three years old and with body weight ≥10–15 kg [148,149], who:

- have not been previously treated with ART; and
- have been previously treated with ART, have no DRV resistance mutations (DRV-RAMs) and have HIV-1 plasma RNA <100,000 copies/mL and CD4 + cell count ≥100 cells × 10^6^/L. In deciding to start DRV treatment in these patients previously treated with ART, the administration should be guided by genotypic testing [150].

After initiating treatment with DRV, patients are advised not to change the dose, dosage form, or discontinue treatment without talking to their healthcare professionals. The drug interaction profile of DRV depends on the use of RTV or COBI as a pharmacokinetic enhancer. Therefore, DRV may have different contraindications and recommendations for concomitant use depending on the use of RTV or COBI for potentiation [151].

### 8.3. Pharmacodynamic Properties. Therapeutic Efficacy and Safety

**Figure 7:**
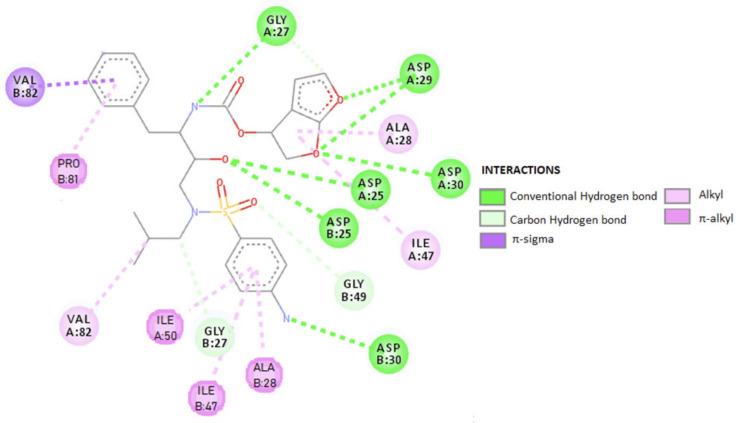
2D diagram representing crystal structure of wild type HIV-1 protease in complex with darunavir (Discovery Studio Client v19. 1.0. 18287). Legend: glycine (GLY27, GLY49); alanine (ALA28); aspartic acid (ASP25, ASP29, ASP30); isoleucine (ILE47, ILE50); proline (PRO81); valine (VAL82); A and B are the chains.

**Figure 8:**
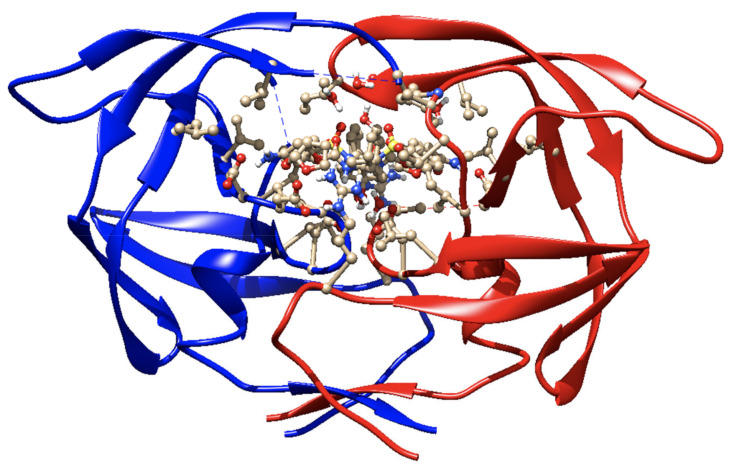
3D structure of DRV-HIV-1 proteases complex (UCSF Chimera version 1.14 (build 42091)). Legend: Blue – chain A, Red– chain B.

The efficacy and safety of DRV alone or in combination with potentiating drugs have been demonstrated in numerous studies [54,152,153,154,155,156]. The results showed that DRV significantly improved virological and immunological outcomes compared to other PIs and was generally well tolerated, with low cytotoxicity. The drug was developed to have a high genetic barrier against the development of resistance and has activity against wild-type HIV-1 and a wide range of viruses resistant to other PIs. DRV has a high affinity for HIV-1, with a binding constant of 4.5 × 10–12 mol/L, in vitro, binding tightly and predominantly to the substrate coating [149] with rigid and flexible molecular docking that allows it to form a very stable complex with the enzyme. This binding is shown in 2D form in Figure 7, and in 3D form in Figure 8 [157]. The molecular docking was done similar to RTV. Following the analysis of the 2D diagram (Figure 7), it is revealed that DRV binds to the following amino acids: glycine (GLY49), alanine (ALA28), aspartic acid (ASP29 and ASP30), valine (VAL82), asparagyne (ASN25), isoleucine (ILE47 and ILE50), and proline (PRO81). The common amino acid ASP30 forms H bonds at the level of the B protein chain. The amino acids ALA28 and ILE47, present at the level of both chains, make alkyl/π–alkyl bonds; VAL82 binds through alkyl and π–sigma. In chain A, ALA28 binds through an alkyl bond, ASP29 and ASP30 through the H bond, and ILE50 through a 3-ol hexadrofuro-furan via π–alkyl bond to the DRV structure. Regarding the B chain, the 4-aminophenyl group, within the protein, establishes H and π–alkyl bonds, with the ASP30 and ILE47 amino acids of the receptor structure. PRO81 forms (via the pyrrolidine protein) a bond through the benzene ring. GLY49 and GLY27 form C–H bonds with the ligand.

Table 3 schematically shows the ligand–receptor interactions, HIV-DRV protease from the A and B chains of the viral protease, the distances between them and the types of bonds that are formed.

If the affinity for wild-type strains is increased, species that have developed resistance to PIs are less sensitive to DRV, and ARV activity is compromised [158].

### 8.4. Drug Resistance

The major complication of ART is the evolution of drug-resistant viral strain variants. This resistance occurs due to rapid viral replication. Most PIs select viral species with one or more specific mutations in the coding region that confer resistance. DRV appears to have a high genetic barrier against developing viral resistance in the case of wild-type virus as well as a large group of clinical isolates resistant to other PIs [159]. Following in vitro studies, it was found that resistance to DRV occurs after more than three years of treatment, and the development of these strains can be suppressed at a drug concentration >400 nmol/mL [149]. Thus, DRV is a viable option for the treatment of both naive and PI treated HIV patients. In addition, the combination of DRV and potentiating drugs significantly reduces the risk of viral resistance, thus contributing to improved efficacy [160,161]. The efficacy and safety of DRV administered alone or in combination therapy have been demonstrated in numerous studies [162,163,164,165]. The results showed that DRV significantly improved the virological and immunological response compared to other PIs and was also well tolerated.

Although structurally, DRV is similar to amprenavir, DRV has two intertwined tetrahydrofuran rings. This difference is important in the antiviral activity of DRV, because the bistetrafuran fragment reverses stereochemistry at the point that binds it to the rest of the molecule and allows additional interactions of DRV with ASP 29, a key amino acid of the HIV protease [161].

### 8.5. Pharmacokinetic Properties

Pharmacokinetic studies in which DRV was administered alone showed a bioavailability of 37%. In combination with RTV, a potent CYP3A inhibitor, the plasma concentration increased, the bioavailability reaching 82%. This demonstrates that DRV is sensitive to a presystemic metabolism or to an efflux effect or even to both. Potentiation drugs reduce these effects. According to CPR, DRV is primarily metabolized by CYP3A. RTV and COBI inhibit CYP3A, thereby significantly increasing plasma DRV concentrations [153,160,161,166,167,168,169,170].

#### 8.5.1. Absorption

DRV is rapidly absorbed after oral administration. The maximum plasma concentration in the presence of a low dose of RTV is generally reached within 2.5–4 h [148,149]. The absolute oral bioavailability of a single 600 mg dose of DRV alone was approximately 37% and increased to approximately 82% in the presence of 100 mg RTV twice daily [166]. The effect of overall pharmacokinetic potentiation by RTV was an approximately 14-fold increase in systemic DRV exposure when a single 600 mg dose of DRV was administered orally in combination with RTV 100 mg twice daily. When administered without food, the relative bioavailability of DRV in the presence of a low dose of RTV was 30% lower compared to co-administration with food. Therefore, DRV tablets should be administered with RTV and food. The type of food does not affect ARV exposure [160,161].

P-glycoprotein from intestinal epithelial cells may decrease oral absorption of PIs by mediating drug efflux. Low levels of intestinal absorption, together with CYP 450 activity, are major factors in the low bioavailability of DRV. The combination of a boosting drug inhibits both the action of CYP 450 and the efflux transport systems in the intestinal mucosa, thus improving the bioavailability of DRV [148,149].

#### 8.5.2. Distribution

DRV is strongly bound to plasma proteins in a proportion of approximately 95%, binding in particular to plasma α1-acid glycoprotein [153,167]. It can also be detected in cervical-vaginal fluid [171], sperm [163,172], and cerebrospinal fluid [13,173,174]. Following intravenous administration, the volume of distribution of DRV alone was 88.1 ± 59.01 (mean ± DS) and increased to 131 ± 49.9 l (mean ± DS) in the presence of 100 mg RTV twice daily.

#### 8.5.3. Drug Metabolism

In vitro experiments with human liver microsomes (HLM) indicated that DRV mainly undergoes oxidative metabolism. DRV is extensively metabolized by the hepatic system CYP 450 and almost exclusively by the CYP3A4 isoenzyme. In humans, at least three oxidative metabolites of DRV were identified, with 10-fold lower activity on the wild-type HIV than the parent substance [149,161].

A study conducted in 2009 [175] demonstrated that RTV, used as a pharmacokinetic enhancer, significantly alters DRV metabolism. In this study, un-boosted DRV was metabolized by carbamate hydrolysis, aliphatic isobutyl hydroxylation, aniline-aromatic hydroxylation and, to a lesser extent, by benzyl-aromatic hydroxylation and glucuronidation. Boosting DRV with RTV resulted in a strong inhibition of carbamate hydrolysis, aliphatic isobutyl hydroxylation, and aromatic benzyl hydroxylation. The booster did not influence the aromatic hydroxylation of benzyl in any way but increased the elimination of glucuronide metabolites. However, the percentage of DRV available for action between RTV-boosted and un-boosted patients was significantly different (48.8%) compared to only 8%, as a result of inhibition of DRV metabolism.

#### 8.5.4. Clearance

After a dose of 400/100 mg, DRV with RTV, approximately 79.5 and 13.9% of the administered dose is found in the feces and urine, respectively. Unchanged DRV was approximately 41.2 and 7.7% of the administered dose in feces and urine, respectively. The terminal clearance half-life of DRV was approximately 15 h when combined with RTV. The intravenous clearance of DRV alone (150 mg) and in the presence of a low dose of RTV was 32.8 and 5.9 L/h, respectively [148,149].

### 8.6. Pharmaco-Toxicology

DRV is generally well tolerated in humans. The most common side effect is rash 7% of cases. This is followed by diarrhea (2.3%), headache (3.8%), abdominal pain (2.3%), constipation (2.3%), and vomiting (1.5%). DRV can also cause allergic reactions, and people allergic to RTV can also have a reaction to DRV [150,176]. High blood sugar levels, diabetes or worsening diabetes symptoms, muscle aches, tenderness or weakness, and increased bleeding in people with hemophilia have been reported in patients taking PIs such as DRV. Changes in body fat have been observed in some patients taking HIV medications including loss of fat from the legs, arms, and face, increased fat in the abdomen and other internal organs, and enlargement of the breasts and fat on the back of the neck [177,178].

As common side effects of DRV are as follows: diarrhea, vomiting, nausea, indigestion, eczema, itching, abdominal pain, bloating, flatulence, headache, fever, peripheral neuropathy, lipodystrophy, insomnia, weakness, fatigue, diabetes, increased creatinine levels, cholesterol, and liver enzymes. Rare side effects (like abnormal liver function, changes in heart rate) may also occur. It must not be used in patients with severe hepatic impairment and should be used with caution in cases of moderate hepatic impairment. Table 4 shows the effects that may occur when co-administering DRV with COBI or RTV [179].

### 8.7. Drug–Drug Interactions (DDI)

**Figure 9:**
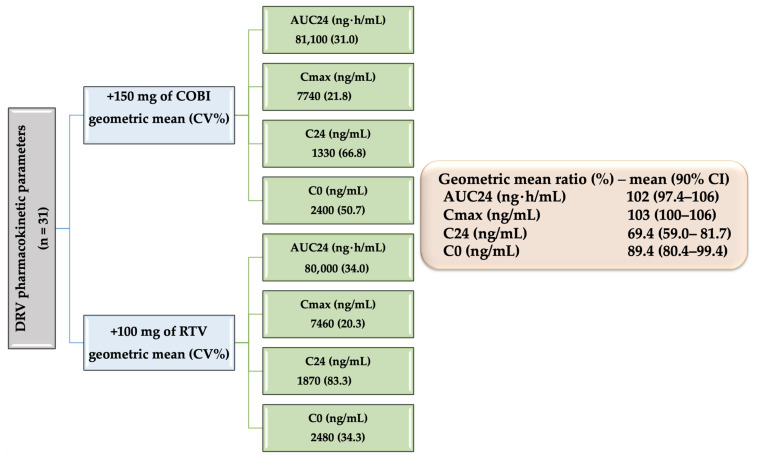
Comparison of pharmacokinetics of 800 mg DRV when co-administered with 150 mg COBI or 100 mg of RTV. Legend: AUC—area under the curve (area under the plot of plasma concentration of a drug vs. time, after dosage); C0—initial concentration; C24—concentration at 24 h; Cmax—maximum concentration; CV—coefficient of variation.

Drug–drug interactions are a permanent concern for clinicians treating HIV-infected patients. In addition to HAART, which requires combinations of multiple ARV drugs, patients should frequently take other medications to prevent or treat opportunistic infections, associated diseases, and to manage ARV side effects. Many of these drug interactions can lead to dose adjustments or even contraindications to their use [187]. However, there are also interactions sought in practice such as that with enzyme inhibitors, used to boost the effects of PIs. CYP 3A4 is the key to metabolism for both DRV and RTV, so their plasma concentrations can be increased by co-administration of enzymatic inhibitors of this isomorph and decreased with CYP 3A4 inducers [188]. Figure 9 compares the pharmacokinetics of co-administration of 800 mg DRV with 150 mg COBI or 100 mg RTV.

## 9. Conclusions and Future Directions

The emergence of PIs was an important step in the treatment of HIV-infected patients. Their use, together with two nucleoside analogues, has radically changed, in a positive way, the life prognosis of patients. If the initially developed PIs had many side effects and thus imposed limitations in administration, recent generations are well tolerated, significantly improving the quality of life of the patients. Today, PIs have a convenient dosage, are administered in a single dose, daily, with high potency and a favorable safety profile. Co-administration of a pharmacokinetic enhancer has greatly contributed to this effective and safe therapeutic profile of PIs. To this, it is added the increased barrier to the occurrence of viral resistance of PIs and the specific immune benefit of this class. All these features make PIs key agents in the fight against HIV. Dual therapeutic regimens were initially designed to be administered to patients in whom monotherapy had failed. However, the practice has proved unquestionable qualities; in addition to the much higher potency, there has been better patient compliance due to the decrease in the pill burden and the reduction in the number and intensity of side effects.

DRV is part of the new generation of PIs, bringing a great advantage in the treatment of HIV/AIDS because it has superior efficacy on wild-type HIV but also on already resistant strains. DRV co-administered with a booster—RTV or COBI—has a higher pharmacokinetic profile due to booster inhibition of metabolic pathways. Boosted DRV is well tolerated by patients, with fewer side effects than other regimens. Therefore, DRV co-administered with RTV or COBI is a viable treatment option for both naive and experienced patients.

COBI and RTV are considered equivalent pharmacokinetic stimulants with similar inhibitory effects on CYP3A4, CYP2D6, P-gp, and other transporters. Similar exposures to elvitegravir, atazanavir, and DRV are made when combined with either substance. However, in the presence of inducers, etravirine, rifamycin, or anticonvulsants, COBI may not be as effective as RTV in inhibiting CYP3A4. However, COBI is a more specific inhibitor of CYP3A than RTV and has no inducing properties. Thus, it is anticipated that drugs metabolized primarily by CYP1A2, CYP2B6, CYP2C8, CYP2C9, and CYP2C19 or subject to glucuronidation reactions will be affected differently by RTV and COBI. Additionally, data on drug–drug pharmacokinetic interactions for COBI are often extrapolated from RTV. However, separate studies are needed in the case of COBI. Although they have similar profiles, problems can also occur when changing a treatment regimen that contains RTV with another that includes COBI. Clinicians need to know the differences in the pharmacokinetic profile of the two boosters, especially when evaluating and managing potential drug interactions, with most HIV+ patients having multiple comorbidities and co-medications. In these situations, monitoring the therapy, by various means, is absolutely necessary in order to be able to act promptly by adjusting the doses or by modifying the therapeutic regimens.

## Figures and Tables

**Table 1:** Ligand–receptor interactions at the A and B chains (Discovery Studio Visualizer).

Amino Acids of Chain A	Ligand-Protein Distance (Å)	Types of Connections	Amino Acids of Chain B	Ligand-Protein Distance (Å)	Types of Connections
ALA28	4.43	π-alkyl	ALA82	4.38	Π–alkyl
PRO81	4.39		PRO81	5.47	
ILE47	4.90		ILE84	5.37	
ALA82	4.35			4.24	Alkyl
	4.98		ALA28	4.24	
	4.26	Alkyl		3.81	
ILE50	4.43		VAL32	4.97	
GLY48	3.68	C-H		5.39	
ASP30	2.58		GLY27	3.05	H bond
ASP25	2.64	H bond	ASP25	2.71	
	2.79			2.75	
ARG8	3.13	π-cation	GLY48	3.33	
				3.39	C–H
			ASP29	2.96	
				2.80	H bond
			GLY49	3.29	C–H

Legend: GLY—glycine; ALA—alanine; VAL—valine, ASP—aspartic acid; PRO—proline; ARG—arginine, ILE—isoleucine.

**Table 2:** Actions of ritonavir (RTV) and cobicistat (COBI).

COBI	RTV
Similar Actions	
Potency on CYP 3A4 [137]. At therapeutic conc., inhibitory effect on the hepatic organic anions’ transporters of OATP polypeptides and MATE1 [116]. Inhibitory effect on the intestinal transporters P-gp and BCRP, thus increasing the absorption of co-administered substances [138]. Determine a slight increase in SCR and an associated decrease in glomerular filtration rate (reported in patients with RTV or COBI regimen included), mainly due to the inhibition of CR secretion, by inhibition of MATE1, and not due to impaired liver function [116,120,139,140]. Activate more or less PXR (which regulates the expression of various enzymes that metabolize drug substances) [142].	
Different Actions	
More selective, at clinically relevant conc. [139]. No inhibitory effects on CYP 2C8 and slightly inhibits CYP 2D6 [138]. Increases SCR conc. (higher SCR levels than in patients with RTV-containing regimens) [116,120,139,140]. Active transporter in tubular cells, with OCT2; higher SCR levels mentioned above are explained by the fact that COBI preferentially accumulates in tubular cells and reaches conc. capable of inhibiting MATE1 [53,141]. Limited effect on PXR, being unlikely to have an enzymatic induction effect on drug metabolism [143,144,145] Less pill burden (available at single tablet regimen) [146]. Neutral effects on serum lipids [146]. Higher risk of DDIs in association with antithrombotic [146]. Contraindicated in HIV-infected pregnant women [146].	Activates PXR and induces CYP1A2, CYP2B6, CYP2C9, CYP2C19, and glucuronidation enzymes [143,144,145]. Higher potency for CYP 3A inhibition [136,137,146]. Fine tuning of individualized ARV regimens in patients with polytherapy [146]. Detrimental effects on serum lipids [146]. Higher risk of DDIs with non-CYP 3A substrates (considering party drugs as well) [146]. Recommended in HIV-infected pregnant women [146].

Legend: ARV—antiretroviral; BCRP—breast cancer resistance protein; COBI—cobicistat; conc—Concentration; CR—creatinine; CYP—cytochrome; DDI—drug-drug interaction; MATE—multidrug and toxin extrusion (a transporter involved in the tubular secretion of creatinine); OATP—organic anion transport protein; OCT2—organic cation transporter; P-gp—P-glycoprotein; PXR—pregnane X receptor (regulates the expression of various enzymes that metabolize drug substances); RTV—ritonavir; SCR—serum creatinine.

**Table 3:** Ligand–receptor interactions at the A and B chain (Discovery Studio Visualizer).

Amino Acids of Chain A	Ligand-Protein Distance (Å)	Types of Connections	Amino Acids of Chain B	Ligand-Protein Distance (Å)	Types of Connections
ASP30	3.32	H bond	GLY49	3.01	C-H
ASP25	2.92		GLY27	3.31	
ASP29	2.89		ILE47	5.08	π-alkyl
	3.20		PRO81	4.99	
GLY27	3.33	H bond	ALA28	4.11	
	3.67	C-H	VAL82	3.44	π-sigma
ILE50	5.04	π-alkyl	ASP30	3.31	H bound
ALA28	4.60	Alkyl			
VAL82	5.38				
ILE47	5.07				

Legend: GLY—glycine; ALA—alanine; ASP—aspartic acid; ILE—isoleucine; PRO—proline, VAL—valine.

**Table 4:** Adverse effects to boosting Darunavir (DRV) with RTV or COBI.

Adverse Effect/Ref.	DRV/COBI	DRV/RTV
Gastrointestinal [122,150,160,172,173,180,181,182,183,184,185]	^v^ diarrhea 28%, nausea 23% ^c^ vomiting, abdominal pain, abdominal distension, dyspepsia, flatulence, increased pancreatic enzymes ^u^ acute pancreatitis	^v^ diarrhea 14.4% ^c^ nausea, vomiting, abdominal pain, increased pancreatic amylase, increased pancreatic lipase, abdominal distension, dyspepsia, flatulence, increased serum amylase ^u^ pancreatitis, gastritis, gastroesophageal reflux, stomatitis, dry mouth, abdominal discomfort, constipation, increased lipase, belching ^r^ stomatitis, hematemesis, cheilitis, dry lips, loaded tongue ^f^ increase in liver enzymes
Others [122,160,172,180,181,182,183,184,185,186]	^v^ fatigue ^u^ asthenia	^v^ increase: cholesterol 10–25%, LDL 9.12–14.4%, TG 3.9–10.4%, alkaline phosphatase 1–3.9% ^c^ asthenia, fatigue ^u^ fever, chest pain, peripheral edema, redness, general malaise, pain, weight loss or gain, HDL decrease, increase in serum alkaline phosphatase, increase in lactate dehydrogenase. ^r^ chills, xerosis ^f^ hypertension, facial edema, decreased bicarbonate
Dermatological—rashes generally mild and moderate, appear in the first 4 weeks of treatment, are remitted to continuous administration [122,150,160,180,183,185]	^v^ maculopapular rash, papular, erythematous, pruritic, generalized rash, allergic dermatitis—16% ^c^ angioedema, pruritus, urticaria	^c^ macular, maculopapular, papular, erythematous, pruritic rash, pruritus, lipodystrophy—lipo-hypertrophy, lipodystrophy, lipoatrophy ^u^ angioedema, generalized rash, urticaria, night sweats, allergic dermatitis, eczema, erythema, alopecia, hyperhidrosis, Steven-Johnson syndrome, acne, dry skin, nail pigmentation, herpes simplex infection, severe skin reactions accompanied or not by fever and/or increased transaminases ^r^ drug eruptions with eosinophilia, systemic symptoms, erythema multiforme, dermatitis, seborrheic dermatitis, skin lesions, xeroderma ^f^ folliculitis, lipoatrophy, toxic skin rash, drug dermatitis, skin inflammation
Metabolic: high glucose levels, grade 2 (up to 15.4%), grade 3 (to 1.7%), grade 4 (<1%), were reported to co-administer DRV/RTV [122,160,180,181,182,183,184,185,186]	^c^ anorexia, diabetes, hypercholesterolemia, hypertriglyceridemia, hyperlipidemia	^v^ elevated glucose levels up to 15.4% ^c^ hyperlipidemia, anorexia, diabetes, hypercholesterolemia, hyper triglyceridemia ^u^ gout, decreased or increased appetite, polydipsia, hyperglycemia, insulin resistance ^f^ hypoglycemia, hyperuricemia, hypocalcemia, hyponatremia, obesity, hypoalbuminemia
Nervous system [122,180,183,185]	^v^ headache	^c^ headache, diabetic neuropathy, dizziness ^u^ lethargy, hypoesthesia, paresthesia, dysgeusia, attention and memory disorders, drowsiness and vertigo ^r^ convulsions, syncope, agitation, sleep disturbances ^f^ transient ischemic attack, progressive multifocal leukoencephalopathy
Liver [122,150,173,180,181,183,184,185]	^v^ increase in liver enzymes	^c^ increase in alanyl amino-transferase (ALT) and aspartate aminotransferase (AST) ^u^ hepatitis, acute hepatitis, cytolytic hepatitis, hepatic steatosis, hepatotoxicity, increased transaminases, serum bilirubin and gamma-glutamyl transferase ^f^ increase in liver enzymes
Psychiatric [122,180,183,185]	^c^ abnormal dreams	^c^ insomnia. ^u^ depression, disorientation, sleep disturbances, abnormal dreams, nightmares, anxiety, decreased libido, irritability ^r^ confusion, unchanged mood, restlessness
Cardio-vascular [122,180,183,185]		^u^ myocardial infarction, angina pectoris, QT prolongation, ECG prolongation, tachycardia, hypertension ^r^ acute myocardial infarction, sinus bradycardia, palpitations
Blood [122,180,181,183,185]		^u^ thrombocytopenia, neutropenia, anemia, leukopeni.
Kidneys [122,180,183,185]	^c^ increase in blood creatinine	^u^ acute renal failure, renal failure, nephrolithiasis, increased blood creatinine ^r^ decreased renal creatinine clearance ^f^ renal failure
Musculoskeletal [122,180,183,185]	^c^ myalgia	^u^ myalgia, osteonecrosis, muscle spasms, muscle weakness, arthralgia, extremity pain, osteoporosis, increased creatine phosphokinase in the blood ^r^ musculoskeletal stiffness, arthritis, joint stiffness ^f^ osteopenia
Respiratory [122,173,180,183,185]		^u^ dyspnea, cough, epistaxis, sore throat ^r^ rhinorrhea ^f^ nasopharyngitis, hiccups, pneumonia, upper respiratory tract infections
Hypersensitivity reaction [122,180,181,183,185]	^c^ drug hypersensitivity	^u^ drug hypersensitivity
Genitourinary [122,180,183,185]		^u^ proteinuria, bilirubinuria, dysuria, nocturia, pollakiuria, erectile dysfunction ^f^ polyuria
Immunological [122,180,183,185]	^u^ inflammatory syndrome of immune reconstitution	^u^ inflammatory syndrome of immune reconstitution
Endocrine [122,180,181,183,185]		^u^ hypothyroidism, increased levels of thyroid hormone in the blood, gynecomastia
Eye [122,180,183,185]		^u^ conjunctival hyperemia, dry eye sensation ^r^ visual disturbances

Legend: ^v^ very common, ≥10%; ^c^ common, 1–10%; ^u^ unusual, 0.1–1%; ^r^ rare, <0.1%; ^f^ frequency unknown.
